# Gene expression-based modeling of overall survival in Black or African American patients with lung adenocarcinoma

**DOI:** 10.3389/fimmu.2024.1478491

**Published:** 2024-11-11

**Authors:** Bin Zhu, Stephanie S. McHale, Michelle Van Scoyk, Gregory Riddick, Pei-Ying Wu, Chu-Fang Chou, Ching-Yi Chen, Robert A. Winn

**Affiliations:** Massey Comprehensive Cancer Center, Virginia Commonwealth University, Richmond, VA, United States

**Keywords:** lung adenocarcinoma, race disparity, African American, immune response, machine learning, overall survival, expression network

## Abstract

**Introduction:**

Lung cancer is a leading cause of cancer-related deaths worldwide. Black/African American (B/AA) populations, in particular, exhibit the highest incidence and mortality rates of lung adenocarcinoma (LUAD) in the United States.

**Methods:**

This study aims to explore gene expression patterns linked to LUAD in B/AA and case-matched white patients, with the goal of developing predictive models for prognosis. Leveraging RNA sequencing data from The Cancer Genome Atlas (TCGA) database, genes and pathways associated with overall survival (OS) were identified.

**Results:**

The OS-associated genes in B/AA patients were distinct from those in white patients, showing predominant enrichment in immune-related pathways. Furthermore, mRNA co-expression network analysis revealed that OS-associated genes in B/AA patients had higher levels of interaction with various pathways, including those related to immunity, cell-ECM interaction, and specific intracellular signaling pathways. Notably, a potential B/AA-specific biomarker, *C9orf64*, demonstrated significant correlations with genes involved in immune response. Unsupervised machine learning algorithms stratified B/AA patients into groups with distinct survival outcomes, while supervised algorithms demonstrated a higher accuracy in predicting survival for B/AA LUAD patients compared to white patients.

**Discussion:**

In total, this study explored OS-associated genes and pathways specific for B/AA LUAD patients. Further validation and clinical application of these findings are warranted to address disparities and improve outcomes in diverse patient populations.

## Introduction

Lung cancer continues to be one of the leading causes of cancer-related deaths worldwide (1). Moreover, significant differences in incidence and mortality rates have been observed across various racial and ethnic groups (2). Black/African American (B/AA) populations have the highest incidence rate of lung cancer in the United States (2). Previous research has highlighted the importance of understanding the molecular mechanisms underlying the oncogenesis of lung cancer, particularly at the genomic level (3, 4). Despite advances in treatment modalities, racial disparities in survival outcomes persist, emphasizing the need for targeted approaches to address lung cancer in diverse patient populations.

Lung adenocarcinoma (LUAD) is the most common type of lung cancer and is a subtype of non-small cell lung cancer (NSCLC). The landscape of LUAD research has been dominated by studies involving predominantly white and Asian patients (4, 5). Moreover, there is a notable lack of racial and ethnic diversity in cell lines used for lung cancer research (6). Therefore, the lack of representation in the research field and clinical trials result in a limited understanding of the disease’s etiology and progression among B/AA lung cancer patients.

Efforts have sought to address this race disparity in LUAD. For example, higher frequencies of mutations in *JAK2* and *PTPRT* genes have been observed in B/AA LUAD patients compared to European Americans (7). Interestingly, growing evidence suggests that race-related disparities in cancer are also present at the epigenetic level. For instance, differentially expressed genes, e.g., immune system-related genes, have been identified between B/AA and white groups across various types of tumors (8, 9). Therefore, using epigenetic data from a specific racial and ethnic group to model LUAD could be beneficial for precision medicine and has the potential to reduce racial disparities in LUAD therapy.

Previous work has established models to predict the survival of LUAD patients based on the expression of specific gene sets, e.g., metabolism-associated genes (10), tumor microenvironment-associated genes (11), and CDK2-related genes (12). In particular, genes involved in the immune system have been reported in several studies that contribute to predicting prognosis of LUAD patients (13, 14). Furthermore, a high-level expression of immune-related genes is beneficial for longer survival (14). However, none of these studies have specifically focused on B/AA patients to investigate racial disparities in their analyses.

The objectives of this study are to explore gene expression patterns linked to LUAD in B/AA patients and develop predictive models with potential clinical applications for prognosis. Through analysis of RNA sequencing data from The Cancer Genome Atlas (TCGA) database, epigenetic biomarkers and pathways associated with overall survival (OS) at the transcriptional level were uncovered. By analyzing the OS-associated gene expression profiles using unsupervised machine learning algorithms, the B/AA patients were stratified into two distinct groups with significantly divergent survival outcomes. Additionally, survival outcomes among B/AA patients were predicted using supervised machine learning algorithms. Furthermore, all the outcomes for B/AA participants were compared with case-matched white individuals and race disparities were examined.

## Methods

### Data source

RNA-seq data and metadata were downloaded from the TCGA, PanCancer Atlas cohort (https://www.cbioportal.org/), which contains 52 B/AA participants. A case-matched design was performed where the 52 B/AA participants were matched with 52 white participants by choosing the same sex, tumor stage, smoking status, and similar ages. Most outcomes in this study were evaluated using the TCGA cohort. To further assess the association between gene expression levels and OS of lung cancer patients, a second cohort was used (GEO number: GSE101929) (15). This cohort included tumor samples from 16 pairs of B/AA and white non-small cell lung cancer patients, with mRNA expression analyzed through microarray sequencing.

### OS analysis

RNA-seq RSEM values were normalized by the VST method and were classified into two levels, i.e., high and low. The Kaplan-Meier survival curve analysis was performed using the ‘survfit’ function in R. P-values were adjusted by the Benjamini–Hochberg procedure. The association between multiple variables in the metadata and OS was tested by the Cox proportional hazards regression using the ‘coxph’ function in R. The association between each variable in the metadata and OS was tested by the Kaplan-Meier survival curve analysis.

### Pathway enrichment

Pathway enrichment for the OS-associated genes and genes in each module in co-expression network analysis was measured by the ‘compareCluster’ function in R using the KEGG pathway (16). Pathway enrichment for genes correlated with four potential biomarker genes, i.e., *C9orf64*, *CUBN*, *REEP2*, and *MRAS*, was tested using the DAVID online tool (17) and enriched biological processes classified by the Gene Ontology database (18) are shown. P-values were adjusted by the Benjamini–Hochberg procedure.

### Unsupervised algorithms to classify expression profiles of OS-associated genes

RNA-seq RSEM values were normalized by the average RSEM value of seven housekeeping genes, i.e., *ACTB*, *GAPDH*, *HPRT1*, *B2M*, *TBP*, *RPL13A*, and *RPS18*. The expression profiles of OS-associated genes were clustered by PCA analysis using the ‘prcomp’ function in R. Alternatively, Bray-Curtis distance among patients was calculated based on the profiles of the 1589 OS-associated genes. Patients were clustered by the NMDS algorithm using the ‘metaMDS’ function in R. The difference of the expression profiles associated with survival status was measured by a PERMANOVA test using the ‘adnois’ function in R.

### Supervised algorithms to predict survival status

RNA-seq RSEM values were normalized by the average RSEM value of seven housekeeping genes, i.e., *ACTB*, *GAPDH*, *HPRT1*, *B2M*, *TBP*, *RPL13A*, and *RPS18*. Unless otherwise specified, to reduce the risk of overfitting in the modeling process, a repeated 2-fold cross-validation with 100 repeats resampling strategy was applied in all predictions. To compare the performance of different algorithms, genes were selected using Spearman’s correlation, and only genes significantly correlated with survival status were used for the prediction. Five algorithms, i.e., random forest, k-Nearest Neighbors, support vector machines with linear kernel, eXtreme gradient boosting, and boosted generalized linear model, were tested using the ‘train’ function in R with the ‘method’ parameter set as ‘rf’, ‘knn’, ‘svmLinear’, ‘xgbLinear’, and ‘glmboost’, respectively. Two feature selection strategies, i.e., Spearman’s correlation and recursive feature elimination (RFE), were performed using ‘cor.test’ and ‘rfe’ functions, respectively, in R.

### Co-expression network analysis

Spearman’s correlation was employed to measure gene co-expression among VST-normalized RSEM values of all genes. The co-expression network was constructed and visualized using Gephi software (19), applying the ForceAtlas2 algorithm. Modularity analysis was performed using the ‘modularity’ function in Gephi with default parameters to categorize genes into different modules. An overall representation analysis was conducted to assess the enrichment of KEGG pathways in each module using the ‘phyper’ function in R. P-values were adjusted by the Benjamini–Hochberg procedure.

### Differential gene expression analysis

The RSEM values of the RNA-seq data were converted to integers and were input into the DESeq2 software for the differential gene expression analysis (20).

## Results

### Cohort selection

To focus our study on B/AA LUAD patients, we selected the TCGA, PanCancer Atlas cohort (https://www.cbioportal.org/), which contains 52 B/AA participants. A case-matched design was performed where the 52 B/AA participants were matched with 52 white patients by choosing the same sex, tumor stage, smoking status, and similar ages (**Supplementary Table S1**).

The influence of race, age, sex, tumor stage, and smoking on OS probability was systematically tested by the Cox proportional hazards regression (**Supplementary Figure S1A**). The results showed only a significant association between tumor stage I and OS. Alternatively, the Kaplan-Meier survival curve analysis was performed to test the influence of each individual variable on OS, and similarly, only the tumor stage had a significant association with OS (**Supplementary Figures S1B–F**). Although race (21), age (22), and smoking (23) have been reported to impact OS of lung cancer patients, since the cohort size is small, there could be potential sampling bias and randomness that led to different observations compared with results in large-scale epidemiological studies. Diverse experimental designs could also lead to varied outcomes.

### Genes and pathways associated with overall survival

**Figure 1:**
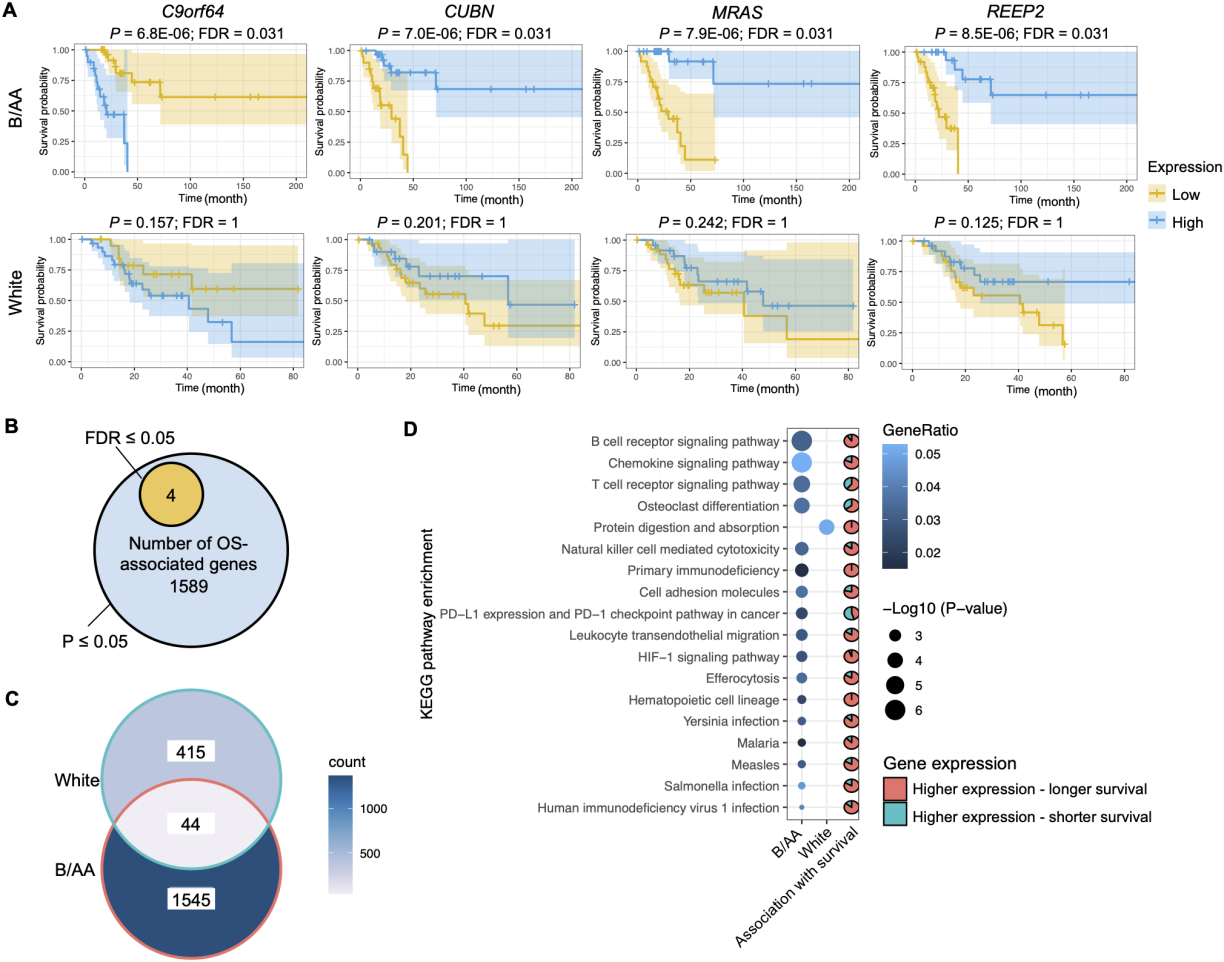
Genes and pathways associated with overall survival. **(A)** The RNA-seq RSEM values were normalized by the variance stabilizing transformation method. Kaplan-Meier survival curve analysis on genes associated with overall survival (OS) in the Black or African American (B/AA) and case-matched white lung adenocarcinoma (LUAD) patients is shown. **(B)** Number of genes that are significantly (P or FDR ≤ 0.05) associated with OS in the B/AA patients. **(C)** Venn diagram illustrating the overlap of OS-associated genes (P ≤ 0.05) between the B/AA and white patients. **(D)** Functional enrichment of OS-associated (P ≤ 0.05) genes in the B/AA and white patients was performed using the ‘compareCluster’ function in R. Significant enrichment (P ≤ 0.01) of KEGG pathways is displayed. The pie chart depicts the proportion of genes within each pathway that exhibit higher expression levels associated with longer survival durations in patients. Specifically, the pie chart for the protein digestion and absorption pathway describes the relationship for the white patients, while all other pie charts are for the B/AA patients.

The RNA sequencing data with RSEM values downloaded from the TCGA database was normalized by variance stabilizing transformation (VST). The VST values were classified into two levels, i.e., high and low, and were utilized to determine the association between gene expression and OS. The Kaplan-Meier survival curve analysis discovered four genes, i.e., *C9orf64*, *CUBN*, *REEP2*, and *MRAS*, with expression significantly (false discovery rate (FDR) ≤ 0.05) associated with OS in the B/AA patients, where high-level expression of *C9orf64* shortened the OS but that of *CUBN*, *REEP2*, and *MRAS* led to longer survival (**Figure 1A**). Furthermore, the four identified potential biomarkers were significantly (P- but not FDR-values ≤ 0.05) associated with OS in both B/AA males and females (**Supplementary Figure S2**) but not in white patients (**Figure 1A** and **Supplementary Data Sheet 1**). Thus, the four genes seem to be potential race- but not sex-specific biomarkers. To further assess the reliability of the four identified biomarkers, their associations with OS was evaluated using the same methodology in an independent cohort case-matched between 16 pairs of B/AA and white LUAD patients [GEO number: GSE101929 (15)]. The results in this second cohort were consistent with those observed in the TCGA cohort (**Supplementary Figure S3**). However, due to the small sample size, the P-values in the second cohort were less significant compared to those in the TCGA cohort. Additionally, since this second cohort was sequenced using microarray technology, *C9orf64* was not included in the dataset.

Limited by the sample size, most FDRs of the association between gene expression and OS in the TCGA cohort were larger than 0.05 (**Supplementary Data Sheet 1**). To explore biological functions associated with OS, the gene set with significant P- but not FDR-values was selected for functional enrichment analysis. Although false-positive results could exist in the analysis outcomes, true-positive results were also included. We identify shared characteristics in the enriched pathways. There were 1589 genes associated with OS (P ≤ 0.05) in the B/AA patients (**Figure 1B**). Surprisingly, 459 genes in the white patients were detected to be associated with OS (P ≤ 0.05), but only 44 OS-associated genes overlapped between the B/AA and white participants (**Figure 1C**).

A functional enrichment analysis on the 1589 B/AA-specific OS-associated genes identified 17 enriched KEGG pathways (P ≤ 0.01). The P-values for the pathway enrichment were insignificant after multiple testing corrections and false-positive outcomes could exist. However, most of the 17 KEGG pathways were related to the immune system, as the top three significantly enriched pathways were B cell receptor signaling, chemokine signaling, and T cell receptor signaling pathways (**Figure 1D**). Other pathways, e.g., *Yersinia*, *Salmonella*, and Human immunodeficiency virus 1 infections, might be enriched because genes involved in these pathways overlapped with immunity-related genes in LUAD. Since a similar trend is observed in these enriched pathways, it seems that the immune system plays an essential role in the OS of the B/AA patients. Furthermore, most OS-associated genes in the pathways had high-level expression in the B/AA participants who survived longer (**Figure 1D**). In other words, a more activated immune system in the B/AAs could prolong the time of survival. The only outlier pathway was the PD-L1 expression and PD-1 checkpoint pathway in cancer, where high-level expression of more than half of the genes were associated with shorter survival. When PD-1 on T-cells binds to PD-L1 or PD-L2 on cancer cells, it inhibits the activity of the T-cells (24). Therefore, the result of the PD-1 pathway is consistent with other observations.

The same functional enrichment analysis was performed on the 459 OS-associated genes in the white patients, which only enriched the protein digestion and absorption pathway (P ≤ 0.01) (**Figure 1D**). This pathway seems to serve as a fundamental process that impacts multiple systems, including the immune system. Previous studies have shown a critical influence of the immune system on LUAD in cohorts of predominantly white patients (13, 14). Thus, these results implied that the immune system in the white patients was statistically less essential than in the B/AA LUAD patients in the case-matched TCGA cohort.

### Modeling of 20-month survival

**Figure 2:**
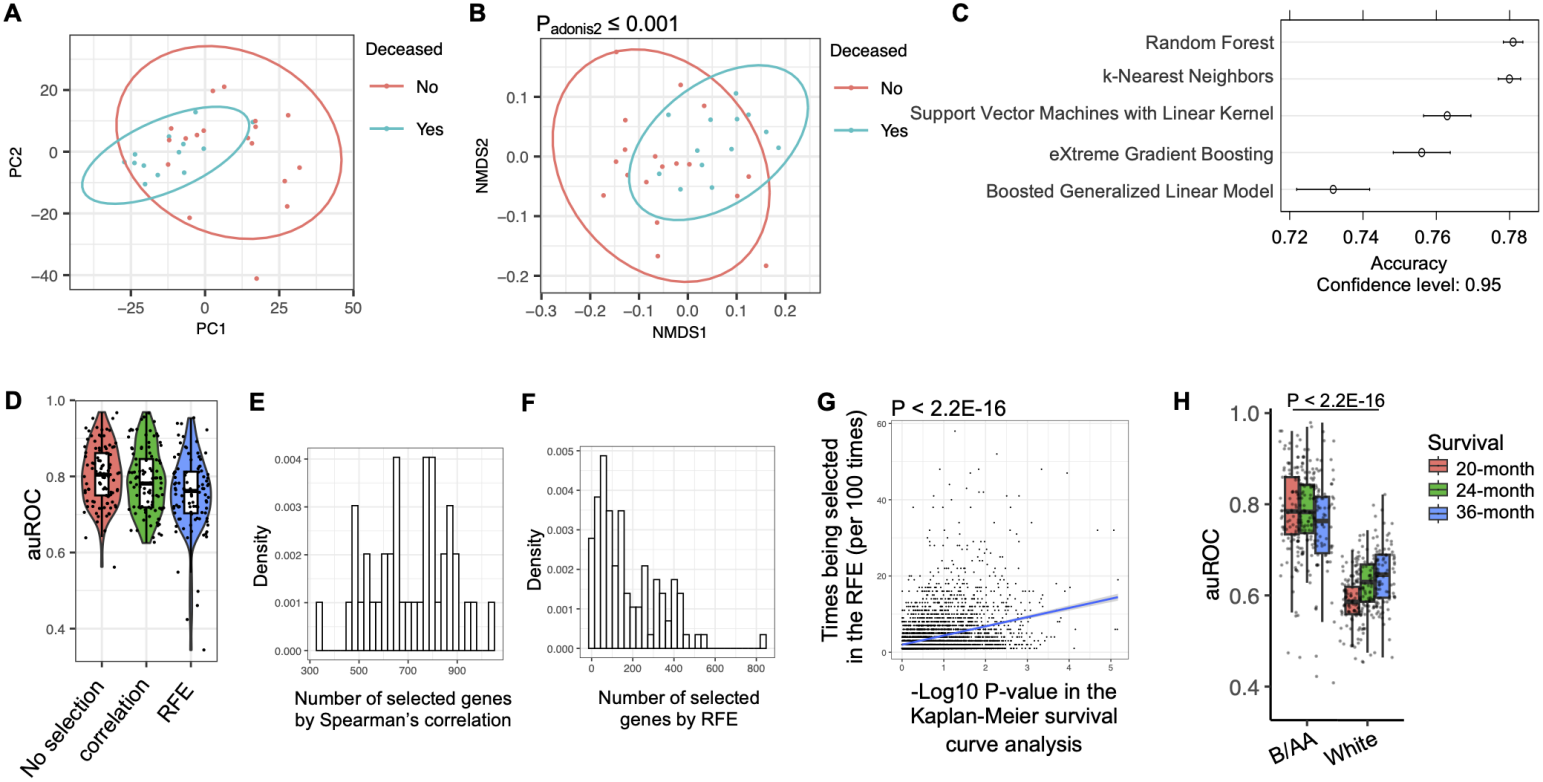
Modeling of LUAD survival. The RNA-seq RSEM values were normalized relative to a set of housekeeping genes (see Methods). **(A)** PCA analysis on the profiles of the OS-associated genes in the B/AA patients is shown. **(B)** Bray-Curtis distance among the B/AA patients was calculated based on the profiles of the 1589 OS-associated genes. The similarity of the patients compared by the NMDS algorithm is visualized. The difference of the expression profiles associated with survival status was measured by a PERMANOVA test using the ‘adnois’ function in R. **(C)** Accuracy comparison of various machine learning algorithms in predicting 20-month survival of the B/AA patients. **(D)** Application of the Random Forest algorithm to predict 20-month survival of B/AA patients using repeated 2-fold cross-validation with 100 repeats. Three feature selection strategies were applied: no feature selection, selection using Spearman’s correlation, and selection using recursive feature elimination (RFE) method. auROC values indicating prediction performance are presented. **(E)** Number of genes selected by the Spearman’s correlation in 100 repeats. **(F)** Number of genes selected by the RFE in 100 repeats. **(G)** Linear relationship between the likelihood of a particular gene being selected in RFE and the P-value of the gene’s association with OS in Kaplan-Meier survival curve analysis quantified by the ‘lm’ function in R. **(H)** Random Forest algorithm with repeated 2-fold cross-validation and feature selection using Spearman’s correlation to predict 20-, 24-, and 36-month survival of B/AA patients, case-matched white patients, white males, and white females, respectively. auROC values and differences between B/AAs and other groups tested by Mann–Whitney U test are presented.

Because similar characteristics associated with OS were observed in the pathway enrichment analysis in the B/AA participants (**Figure 1D**), we classified the B/AA patients by the expression of the 1589 OS-associated genes. The RSEM values were normalized relative to a set of housekeeping genes and subjected to PCA analysis. Most B/AA patients who died after 20 months were separated from the survival patients on the PCA plot (**Figure 2A**). Alternatively, the Bray-Curtis distance among the B/AA patients was calculated based on the normalized RSEM values of the 1589 OS-associated genes, and the similarity of the patients was compared by the NMDS algorithm. Again, the B/AA patients were stratified into clearly distinct groups based on their 20-month survival status (**Figure 2B**). Furthermore, an Adonis test demonstrated a significant difference in the Bray-Curtis distance between the expression profiles of the 1589 OS-associated genes among B/AA patients who survived versus those who were deceased after 20 months.

Various machine learning algorithms were employed to predict the 20-month survival status (see details in the Methods). To mitigate overfitting, a resampling strategy involving repeated 2-fold cross-validation with 100 repeats was utilized. In other words, half of the samples were randomly picked up to train a random forest model and the other half were used to evaluate performance. The process was repeated 100 times. To speed up the training process, only genes significantly associated with the 20-month survival status were selected for the modeling. The performance of the prediction was determined by the accuracy. The results suggested that the random forest algorithm had the best performance (**Figure 2C**). Therefore, the random forest algorithm and the same resampling strategy was applied in the modeling process described below.

Considering potential clinical applications, we aimed to streamline the model using a feature selection procedure to identify the most predictive genes. Initially, when all the genes were utilized for prediction, the median area under the ROC curve (auROC) value across 100 repeats was approximately 0.8 (**Figure 2D**). The first feature selection strategy involved a filter approach employing Spearman’s correlation to retain only genes significantly associated with OS. This approach maintained an average of 700 genes in each prediction (**Figure 2E**). Alternatively, a wrapper approach called recursive feature elimination (RFE) was employed, wherein the model was iteratively trained and less important genes were systematically removed, resulting in an average of fewer than 150 genes retained for modeling (**Figure 2F**). The auROC values were slightly but not significantly reduced by the feature selections (**Figure 2D**). Notably, a linear relationship was observed between the likelihood of a particular gene being selected in the recursive feature elimination and the P-value of the gene’s association with OS in the Kaplan-Meier survival curve analysis (**Figure 2G**). These results indicated that genes more significantly associated with OS were more likely to be selected for predicting 20-month survival.

The frequency of genes selected during the RFE procedure for predicting OS in B/AA LUAD patients is shown in **Supplementary Data Sheet 3**. To further assess the potential clinical utility of the RFE strategy, only 90 genes, selected at least 20 times across 100 individual RFE iterations (**Supplementary Data Sheet 3**), were used to predict OS in these B/AA LUAD patients, employing a Leave-One-Out strategy as previously described (25). The resulting auROC of 0.936 demonstrates strong predictive performance with just 90 genes as input features (**Supplementary Figure S4**). This gene set size is practical for clinical applications, as a custom microarray can efficiently analyze the expression of 90 genes simultaneously.

Overall, the results suggest that the 20-month survival of the 52 B/AA LUAD patients are predictable and genes more significantly associated with OS are more important in the prediction.

Using the same algorithm and resampling strategy, along with all genes, we attempted to predict the survival of the 52 white patients. However, the auROC values obtained were significantly lower compared to the models for B/AA participants (**Figure 2H**). This observation aligns with the findings indicating fewer enriched pathways associated with OS in the white patients, suggesting less shared characteristics in OS-associated gene expression patterns in white than B/AA patients involved in this case-matched cohort.

### mRNA co-expression network in the B/AA LUAD patients

To investigate the roles of the potential OS-associated biomarker genes in the B/AA patients, a gene co-expression network was constructed using Spearman’s correlation among the VST-normalized RSEM values of all genes. In this network, each node represents a gene, and each edge signifies a significant correlation (FDR ≤ 0.05) between two genes. The network encompassed 10,575 genes, each of which exhibited at least one significant correlation with another gene.

**Figure 3:**
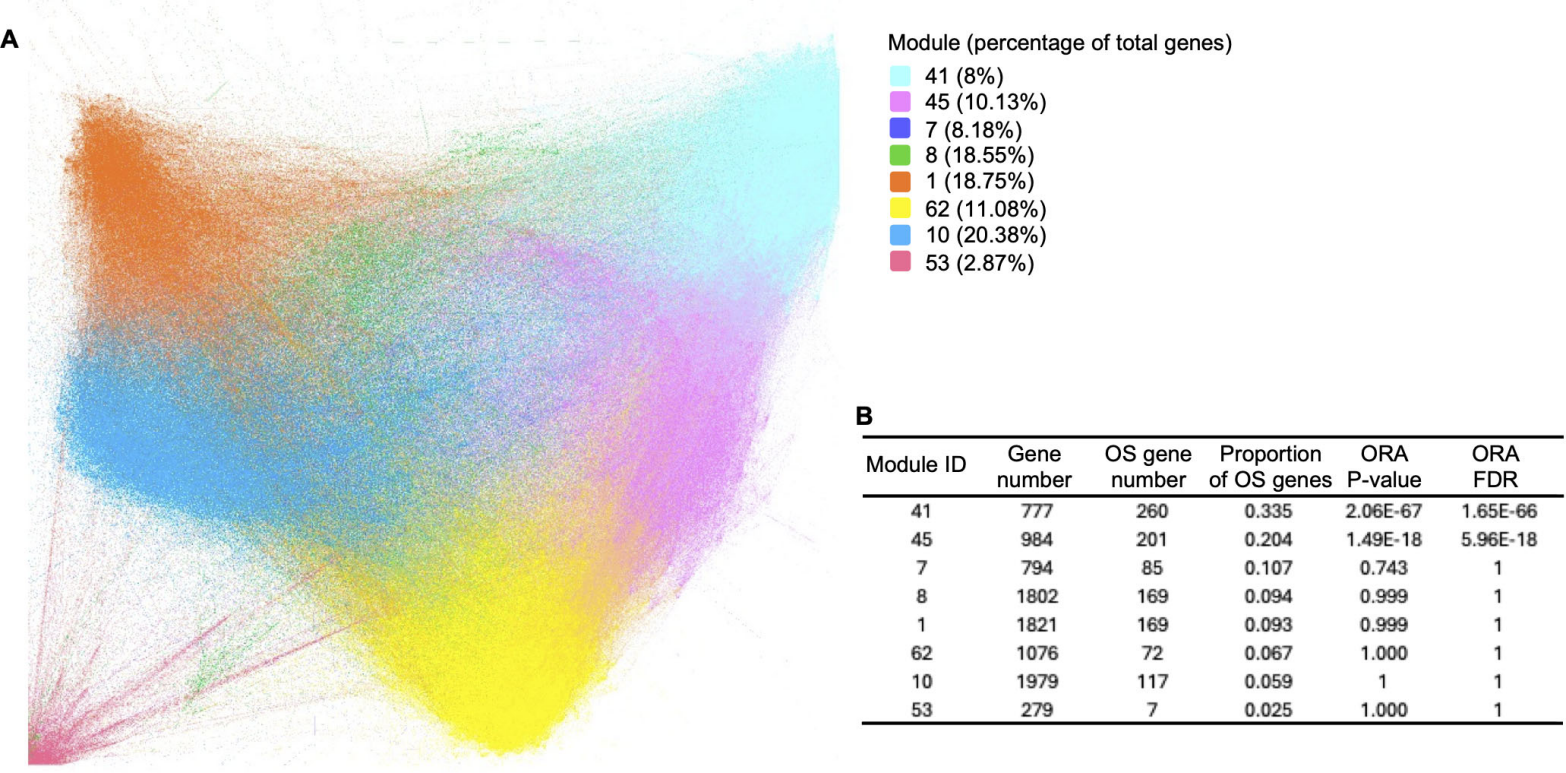
Gene co-expression network in B/AA LUAD patients. **(A)** Spearman’s correlation was employed to measure gene co-expression among VST-normalized RSEM values of all genes. The co-expression network was constructed and visualized using Gephi software, applying the ForceAtlas2 algorithm. Modularity analysis categorized genes into 77 modules. Primary modules containing at least 279 genes in each module are color-coded. **(B)** An overall representation analysis (ORA) was conducted to assess the enrichment of KEGG pathways within the 8 modules. The numbers of total genes and OS-associated genes, along with ORA P-values, are displayed.

The network was analyzed and visualized by the Gephi software (19). Specifically, the network was generated utilizing the ForceAtlas2 algorithm, and modularity analysis was performed to categorize genes into approximately 80 modules (**Figure 3A**). The top 8 primary modules each comprises at least 279 genes, and the proportions of OS-associated genes within these modules are depicted (**Figure 3B**). Conversely, the remaining modules contain a limited number of genes (≤ 16 genes). To assess the enrichment of OS-associated genes within the 8 modules, an overall representation analysis (ORA) was conducted. This analysis revealed that modules 41 and 45 exhibited significantly higher proportions of OS-associated genes (FDR ≤ 0.05). Notably, modules 41 and 45 were also closely situated in the network (**Figure 3A**), suggesting a more robust correlation between these two modules.

**Figure 4:**
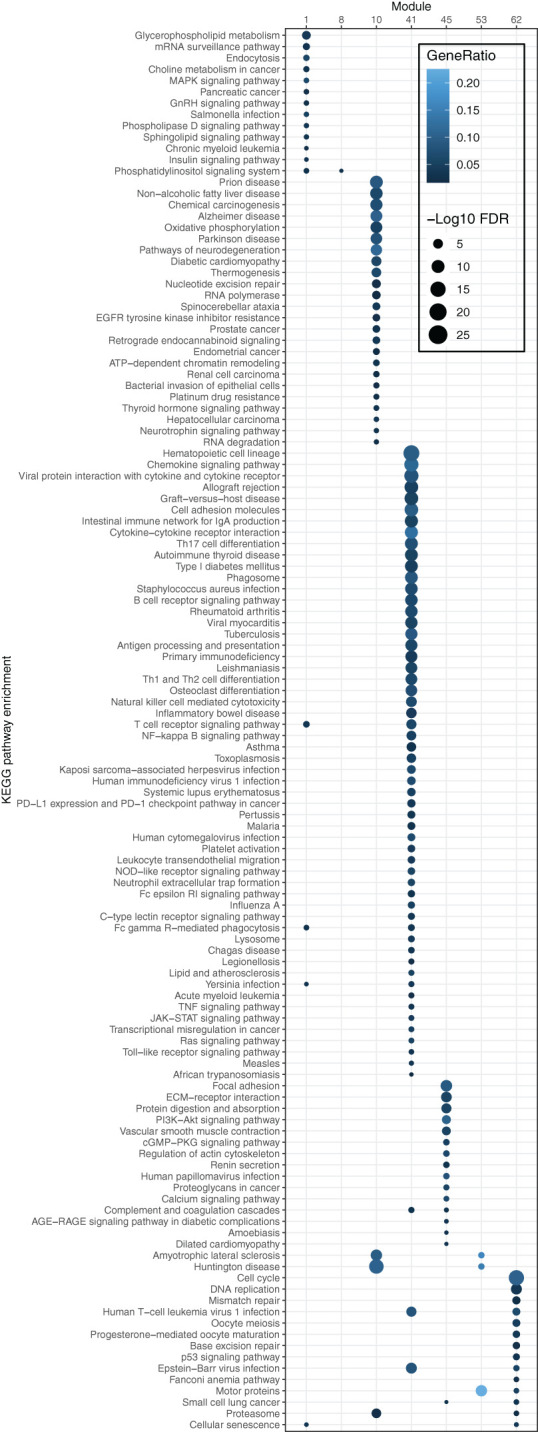
Pathway enrichment of genes in each module of the co-expression network for B/AA LUAD patients. The enrichment was tested by the ‘compareCluster’ function in R. Enriched pathways with FDR ≤ 0.05 are shown.

To elucidate the biological functions of the modules, KEGG functional enrichment analysis was conducted. The results revealed that module 41 was significantly enriched (FDR ≤ 0.05) in pathways associated with the immune system, including the chemokine signaling pathway, intestinal immune network for IgA production, cytokine-cytokine receptor interaction, and Th17 cell differentiation (**Figure 4**). Moreover, most pathways enriched in the OS-associated genes (as shown in **Figure 1D**), e.g., B cell receptor signaling, chemokine signaling, T cell receptor signaling pathways, and natural killer cell-mediated cytotoxicity, overlapped with the pathways enriched in module 41. These findings illustrate that the OS-associated genes not only exhibit functional enrichment in the immune system but also demonstrate a relatively higher degree of correlation with other genes involved in the immune system compared to genes in other modules or with other functions. Thus, the results underscore the significance of the immune system in influencing OS in the B/AA patients.

Module 45 contains 15 enriched pathways (**Figure 4**). The overlapped biological meaning in the 15 pathways revolves around the regulation of cell-ECM interactions, intracellular signaling pathways, and cytoskeletal dynamics, e.g., ECM−receptor interaction pathway, PI3K-Akt and calcium signaling pathway, and regulation of actin cytoskeleton pathway. These pathways have implication in various physiological and pathological processes, including cell adhesion, migration, proliferation, and collectively contribute to the maintenance of cellular homeostasis and the response to extracellular cues in lung cancer (26–28).

### Potential biomarker genes in the mRNA co-expression network for B/AA LUAD patients

**Figure 5:**
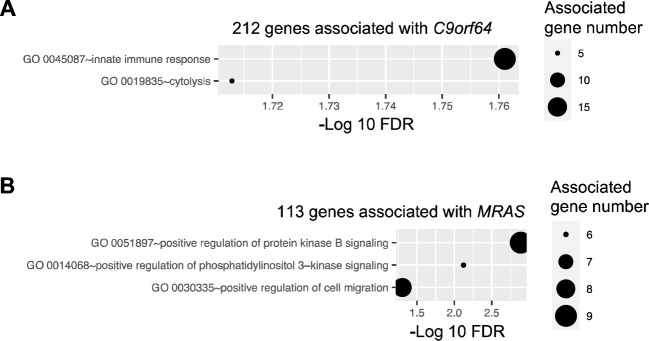
Pathway enrichment of genes correlated with potential biomarker genes. Pathway enrichment analysis was performed for genes correlated with four potential biomarker genes, i.e., *C9orf64, CUBN, REEP2*, and *MRAS*, utilizing the DAVID online tool. Enriched biological processes, classified by the Gene Ontology (GO) database, are depicted for genes correlated with *C9orf64* **(A)** and *MRAS* **(B)**. Notably, no pathways were found to be significantly enriched in CUBN- and REEP2-correlated genes.

*C9orf64* has been reported as an epigenetic biomarker that distinguishes patients with NSCLC from those with nonmalignant lung disease at the transcriptional level (29) and has a higher level of methylation in ovarian cancer (30). However, the biological function of *C9orf64* has not been characterized in previous studies. In this study, *C9orf64* was found to be significantly (FDR ≤ 0.05) correlated with 212 genes. Moreover, these 212 genes exhibited significant (FDR ≤ 0.05) functional enrichment in innate immune response (**Figure 5A**). Therefore, these findings suggest that the expression of *C9orf64* is associated with the expression of immunity-related genes. This implies that *C9orf64* could potentially serve as a biomarker or play a certain role in the immune system in B/AA LUAD patients.

Similar tests were conducted on *CUBN*, *REEP2*, and *MRAS*. Previous studies have indicated that *MRAS* can regulate mitogen-activated protein kinase (MAPK)/extracellular signal-regulated kinase and phosphatidylinositol 3-kinase signaling pathways, thereby modulating various cellular processes related to cell growth, survival, and metabolism (31). Consistent with these findings, enrichment analysis revealed that the 113 genes correlated with *MRAS* in the network were significantly associated with the positive regulation of the protein kinase B and phosphatidylinositol 3-kinase signaling pathways and cell migration (**Figure 5B**). However, the role of *MRAS* in cancer remains obscure (32). Additionally, there was no pathway significantly enriched in genes correlated with *CUBN* or *REEP2*.

### Difference in gene expression between B/AA and white LUAD patients

**Figure 6:**
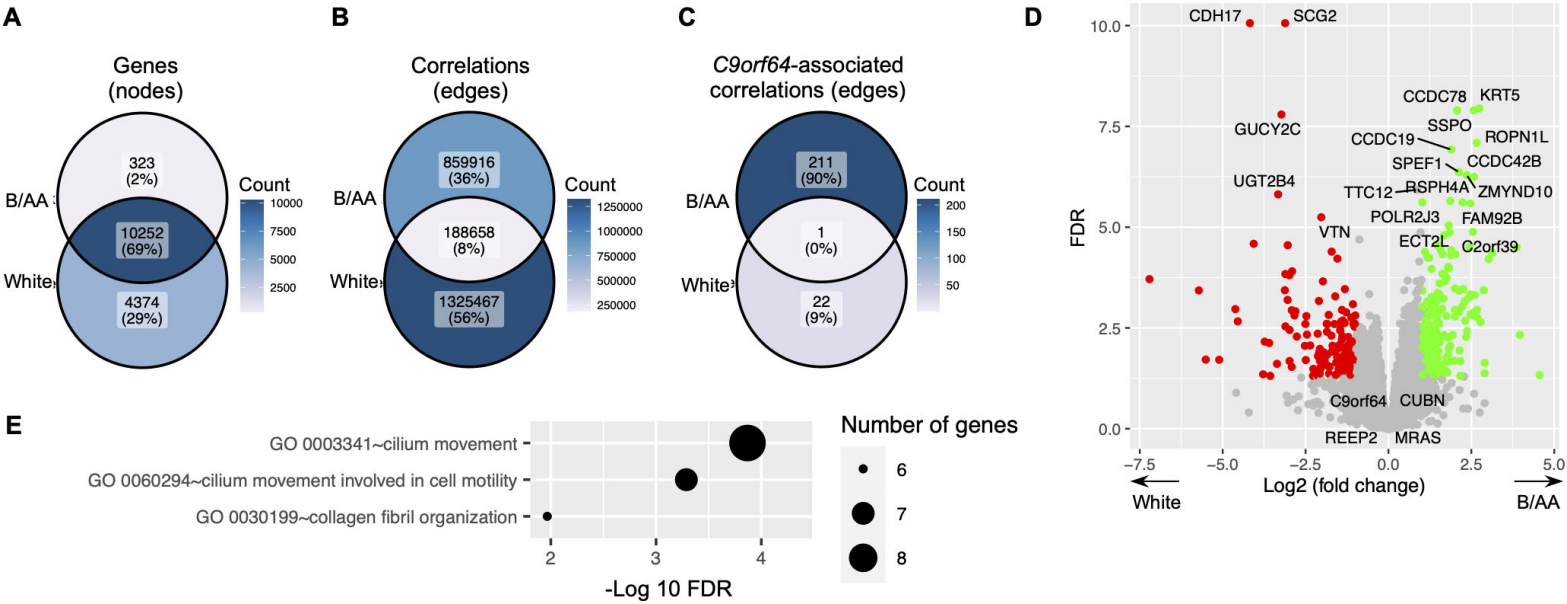
Difference in gene expression between B/AA and white LUAD patients. A comparable gene co-expression network was constructed for the matched white LUAD patients using the same methods. The numbers of total genes **(A)**, significant correlations **(B)**, and *C9orf64*-associated correlations **(C)** in the networks for the B/AA and white patients are compared. **(D)** The differentially expressed genes between the B/AA and white patients tested by DESeq2 are illustrated, where the 20 most significant outcomes and the four identified potential B/AA-specific biomarkers in OS are annotated. **(E)** Pathway enrichment analysis was performed for genes differentially expressed in the B/AA and white patients. Significantly enriched biological processes, classified by the GO database, are depicted.

A comparable gene co-expression network was constructed for the matched white LUAD patients using the same methods. The majority of genes present in the network for B/AA patients also featured in the network for white participants (**Figure 6A**). However, there was only an 8% overlap between correlations in the networks for the B/AA and white patients (**Figure 6B**). For instance, while 23 *C9orf64*-associated genes were identified in the white patient network, only one of these genes correlated with *C9orf64* in the network for the B/AA participants (**Figure 6C**). The immune response pathway exhibited significant enrichment among the 212 *C9orf64*-correlated genes in the B/AA patients (**Figure 5A**). Conversely, there was no significantly enriched pathway among the 23 *C9orf64*-associated genes in the white patient (data not shown). These findings underscore a pronounced racial disparity in gene expression interaction among B/AA and white LUAD patients.

Differentially expressed genes between the B/AA and white LUAD patients were detected by the DESeq2 software (20). The results, including the 20 most significantly differentially expressed genes and the four identified potential B/AA-specific biomarkers in OS, were illustrated in **Figure 6D**. Interestingly, most genes associated with OS did not show differential expression between the two racial groups (**Figure 6D** and **Supplementary Data Sheet 1, 2**). Furthermore, pathway enrichment analysis did not identify any biological functions (**Figure 6E**) that overlapped with OS-associated pathways (**Figure 1D**). These results suggest that the racial disparities highlighted earlier may not stem from differences in gene expression levels but rather from divergent interactions among gene expressions.

## Discussion

The study’s cohort selection addresses a crucial gap in lung cancer research by focusing on B/AA LUAD patients, a demographic historically underrepresented in clinical studies. The identified potential biomarker, *C9orf64*, exhibited significant correlations with genes enriched in immune system-related pathways. Moreover, the 1589 OS-associated genes (P ≤ 0.05) demonstrated functional enrichment in the immune system. Additionally, these OS-associated genes were notably enriched in module 41 during network analysis, where module 41 also displayed functional enrichment in the immune system. Collectively, these findings underscore the influence of the immune system on OS outcomes in B/AA LUAD patients.

The activation of the OS-related immune pathways identified in the B/AA patients can lead to better immune responses, more efficient destruction of cancer cells, and improved control of tumor growth and metastasis. Several pathways, such as T cell receptor, B cell receptor, and natural killer cell signaling, directly enhance immune surveillance and tumor cell killing (33). Others, like efferocytosis, chemokine signaling and leukocyte transendothelial migration, support the recruitment and activation of immune cells to the tumor site (33).

Previous studies have demonstrated the prognostic significance of immune-related biomarkers in lung cancer (13, 14), including the role of immune checkpoint inhibitors in improving outcomes in certain patient subgroups (34). Particularly, several cytokines are specific biomarkers for lung cancer diagnosis in B/AA patients (35). The findings of this study further support the potential prognostic and therapeutic implications of targeting immune-related pathways in LUAD patients. It is worth noting that race disparities between B/AA and white individuals in LUAD have been demonstrated in this study, with evidence suggesting that more immune-related genes and pathways are associated with OS in B/AA patients. Consequently, utilizing genes involved in these immune pathways for LUAD prognosis and immune checkpoint inhibitors for lung cancer treatment could potentially be more effective in B/AA patients. In previous studies primarily comprising white participants (13, 14), the influence of the immune system on the survival of LUAD patients has been established. Consequently, the comparatively weaker association observed between the immune system and OS in the white patients within this study may stem from a smaller sample size in the case-matched design.

Although different patterns in gene co-expression networks were observed between the B/AA and white LUAD patients, the mechanism by which immune-related genes exert a more pronounced influence on the survival of the B/AA patients remains obscure. Consistent with our findings, two studies demonstrate enhanced efficacy of immunotherapy in prolonging survival among advanced NSCLC patients who are non-Hispanic Black (36) or of African ancestry (37). However, another study has not identified a significantly higher survival rate in B/AA NSCLC patients compared to white patients who have received immunotherapy (38). Nevertheless, it is evident that B/AA patients face significantly lower odds of receiving immunotherapy (39). Therefore, the role of the immune system may be more critical for survival in LUAD patients who have a lower chance of receiving immunotherapy, such as B/AA patients. The lower odds of receiving immunotherapy could be associated with an averagely lower socioeconomic status of B/AA population (40, 41). Hence, the lack of information on immunotherapy as well as other potential impact factors, e.g., socioeconomic status, education, and comorbidities in this cohort, limited the exploration of mechanisms of race disparity in this study. Further investigation is warranted to elucidate the mechanisms underlying the observed disparities with more clinical and demographical metadata.

The mechanism leading to the different patterns in gene co-expression networks is also unclear. Previous studies have shown both genetic (7) and environmental (42) differences, e.g., different SNPs and socioeconomic status, between B/AA and white populations. The association between these factors and gene co-expression patterns, as well as whether gene co-expression patterns mediate the influence of the aforementioned factors on LUAD, remains to be determined.

Overfitting may be a concern given the small cohort size. To mitigate this issue, a repeated 2-fold cross-validation with 100 repetitions was employed during the modeling process. Specifically, the average auROC approached 80% across 100 individual tests, which suggested that such modeling strategies, i.e., the random forest algorithm, normalization method using housekeeping genes, and RFE procedure, was reliable in this cohort. Although the small cohort lacks full representation of the entire LUAD population, pathway enrichment analyses revealed shared gene expression characteristics among B/AA LUAD patients, and the modeling auROC values demonstrated that the OS of B/AA LUAD patients was predictable using RNA sequencing data. Therefore, these modeling strategies could have potential applications in larger cohorts to evaluate their clinical utility in guiding treatment decisions.

To enhance the feasibility of the pipeline for clinical application, the RNA-seq data were normalized using housekeeping genes in predictive modeling. Similar prediction accuracy was obtained when the RNA-seq data were normalized by the VST method (data not shown). Additionally, given that, on average, fewer than 200 genes were required for prediction, qPCR or microarray could be a viable alternative to RNA-seq, ensuring accurate prediction in clinical settings.

To simplify the model for clinical application, other types of data such as methylation or copy number alteration were not included in the analysis. Instead, metadata such as age and smoking status, which were readily available in clinical settings, were merged with the RNA-seq data in the modeling process. However, the inclusion of metadata did not significantly impact the quality of the models (data not shown). A plausible reason for the lack of impact could be that these factors were not significantly associated with OS within this particular cohort. It remains possible that these metadata may prove beneficial for prediction in another cohort where they may exhibit a stronger association with OS.

The mRNA co-expression network analysis offers valuable insights into the biological functions of OS-associated biomarkers and their interactions with other genes in B/AA LUAD patients. The function of the uncharacterized gene, *C9orf64*, was inferred by analyzing the functions of its co-expressed genes within the network. Given the specificity of the network for B/AA patients, it holds potential for studying other uncharacterized race-specific gene interactions in the B/AA population. This approach could facilitate a deeper understanding of genetic mechanisms underlying disease outcomes in this demographic group.

Overall, findings in this study contribute to a deeper understanding of the mechanisms underlying OS in B/AA patients with LUAD. By elucidating potential biomarkers, pathways, and gene co-expression networks associated with survival outcomes, this research lays the groundwork for future studies aimed at improving personalized treatment strategies and addressing disparities in LUAD outcomes among different racial and ethnic groups.

## Data Availability

Publicly available datasets from TCGA were downloaded from the cBioPortal (https://www.cbioportal.org/). The dataset for the GSE101929 cohort was downloaded from the Gene Expression Omnibus (https://www.ncbi.nlm.nih.gov/geo/query/acc.cgi?acc=GSE101929).
