# Supplementary material for: Gene expression-based modeling of overall survival in Black or African American patients with lung adenocarcinoma
Source: Front Immunol. 2024 Nov 11;15:1478491. doi: 10.3389/fimmu.2024.1478491 (PMC11586367; doi:10.3389/fimmu.2024.1478491)
Supplement: Supplementary file 4 [file Presentation1.pdf]

A

	coef	exp(coef)	se(coef)	z	Pr(>|z|)
RACE	-0.29086	0.74762	0.36348	-0.8	0.424
AGE	0.0173	1.01745	0.02194	0.788	0.43
SEX	-0.35651	0.70012	0.40519	-0.88	0.379
AJCC PATHOLOGIC TUMOR STAGE1	-1.86348	0.15513	0.79476	-2.345	0.019 *
AJCC PATHOLOGIC TUMOR STAGE2	-0.0652	0.93688	0.79819	-0.082	0.935
AJCC PATHOLOGIC TUMOR STAGE3	-0.77679	0.45988	0.90739	-0.856	0.392
AJCC PATHOLOGIC TUMOR STAGE4	NA	NA	0	NA	NA
TOBACCO SMOKING HISTORY INDICATOR2	0.35291	1.4232	0.83538	0.422	0.673
TOBACCO SMOKING HISTORY INDICATOR3	1.1582	3.18421	0.85359	1.357	0.175
TOBACCO SMOKING HISTORY INDICATOR4	0.5271	1.69402	0.79818	0.66	0.509
TOBACCO SMOKING HISTORY INDICATOR5	2.10021	8.16789	1.30129	1.614	0.107

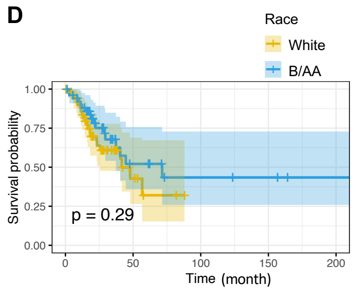

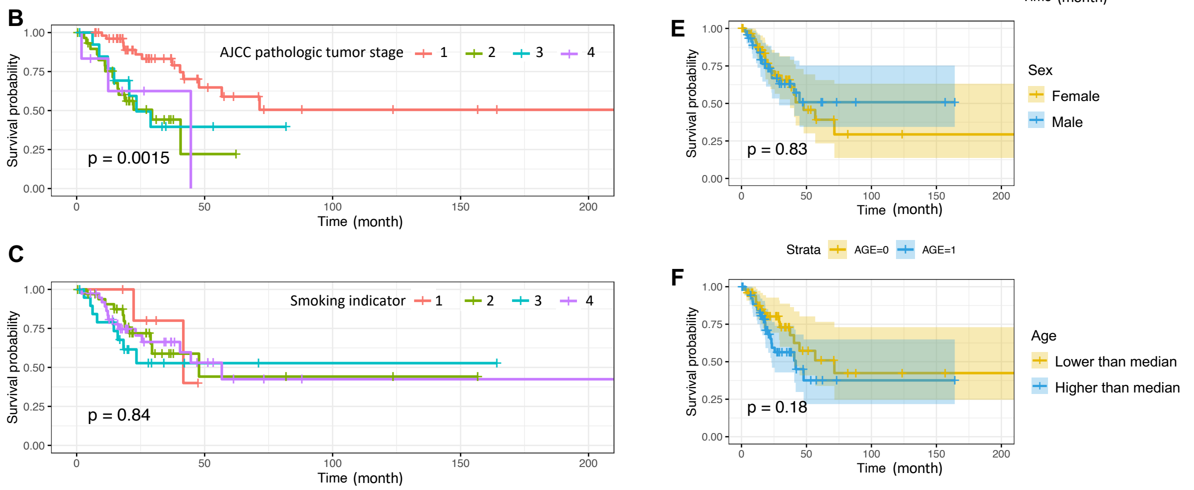

Figure S1 Association between metadata and overall survival (OS). (A) The association of multiple variables in the metadata with OS measured by the Cox proportional hazards regression. **(B-F)** The association of each variable in the metadata with OS measured by the Kaplan-Meier survival curve analysis.

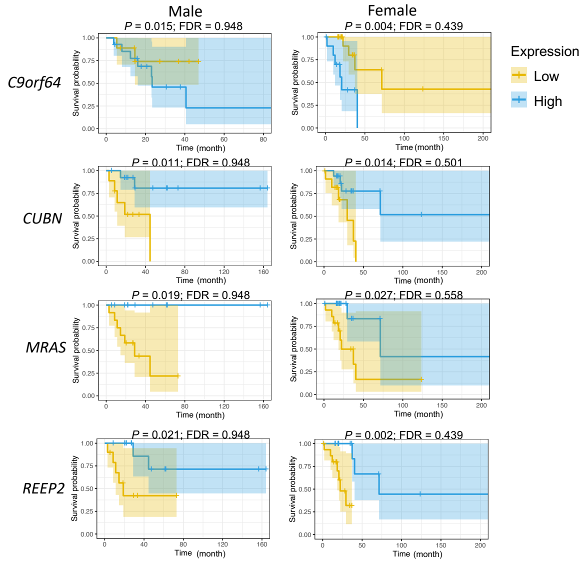

Figure S2 Kaplan-Meier survival curve analysis to test the association between four biomarker genes and OS in the B/AA males and females.

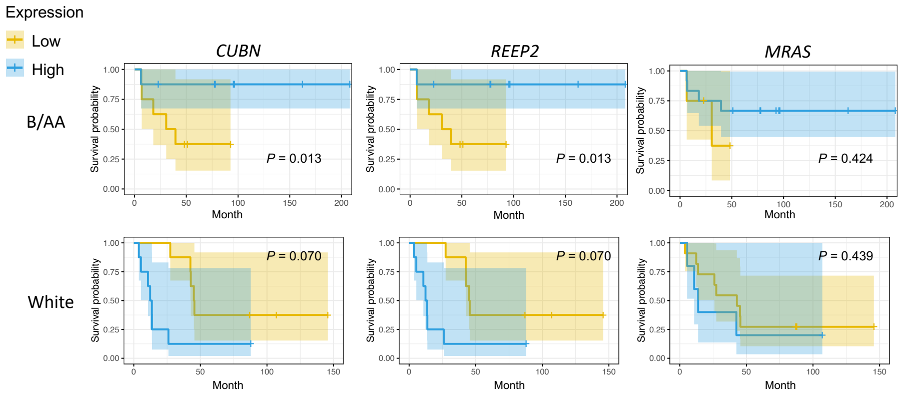

Figure S3 Kaplan-Meier survival curve analysis on genes associated with overall survival (OS) in the Black or African American (B/AA) and case-matched white lung adenocarcinoma (LUAD) patients. The microarray values in the cohort (GEO number: GSE101929) were normalized by the variance stabilizing transformation method.

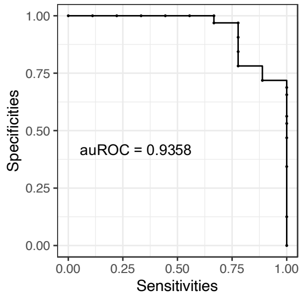

Figure S4 Prediction of OS in B/AA LUAD patients using selected genes. There were 90 genes selected at least 20 times across 100 individual RFE iterations as shown in Figure 2D and Supplementary Dataset 3. These genes were utilized to predict the OS of the B/AA LUAD patients using an Leave-One-Out strategy and the auROC curve was shown.

Table S1 Clinical and demographic characteristics of the case-control study subjects.

	B/AA	White	P-value
Age			0.26
Mean (SD)	60.0 (10.3)	62.3 (9.0)	
Range	39-80	42-81	
Sex (%)			1
Male	23 (45%)	23 (45%)	
Female	26 (55%)	26 (55%)	
Tobacco smoking history indicator			1
1	3 (5.8%)	3 (5.8%)	
2	17 (32.7%)	17 (32.7%)	
3	10 (19.2%)	10 (19.2%)	
4	19 (36.5%)	19 (36.5%)	
5	1 (1.9%)	1 (1.9%)	
AJCC pathologic tumor stage			1
1	27 (51.9%)	27 (51.9%)	
2	15 (28.8%)	15 (28.8%)	
3	7 (13.5%)	7 (13.5%)	
4	3 (5.8%)	3 (5.8%)	
